# Gestational diabetes mellitus and disordered eating behaviours: A qualitative study of online forum discussions

**DOI:** 10.1111/dme.70322

**Published:** 2026-04-17

**Authors:** Yasaman Noori, Emilie Cote, Shaneh Clark, Hayden Chambers, Madeleine Benton

**Affiliations:** ^1^ Department of Psychological Medicine Institute of Psychiatry, Psychology and Neuroscience, King's College London London UK; ^2^ Department of Women and Children's Health School of Life Course & Population Sciences, King's College London London UK

**Keywords:** disordered eating, gestational diabetes mellitus, maternal health, pregnancy, qualitative

## Abstract

**Aims:**

To explore how gestational diabetes mellitus (GDM) and disordered eating behaviours (DEBs) in pregnancy and postpartum are discussed on an online forum.

**Methods:**

A qualitative reflexive thematic analysis of threads and comments from publicly accessible Reddit forums. Posts were included if they referred to current or previous GDM and described experiences related to DEBs. Only English‐language posts were analysed, with no geographical restrictions.

**Results:**

A total of 144 posts referring to DEBs in the context of current or previous GDM were analysed. Three overarching themes were developed: (1) GDM management as a trigger for DEBs, capturing how clinical demands around diet and glucose monitoring intensified preoccupations with food and control; (2) Multilayered distress impacting on relationship with food and disordered eating, highlighting how feelings of shame, unpredictability of GDM, and perceived blame compounded food‐related anxiety and DEBs; (3) Reclaiming agency—disclosure, coping, and adaptive strategies, capturing how some women regained feelings of control by sharing experiences, using adaptive coping strategies, and engaging with online peer support.

**Conclusions:**

GDM care protocols may exacerbate or precipitate DEBs due to their focus on dietary surveillance, glucose control and fear‐based messaging. Individualised, evidence‐informed care that considers psychological experiences across pregnancy and postpartum could improve both clinical and psychological outcomes for women.

**What's new?**

- Disordered eating has been documented in T1DM and T2DM. Pregnancy is a uniquely vulnerable period for eating‐related distress, but little is known about how GDM management may contribute to the emergence or reactivation of disordered eating behaviours.
- Women describe GDM management as intensifying preoccupation with tracking, moralised food messaging, and distress around glucose targets, contributing to the onset or relapse of disordered eating behaviours during pregnancy and postpartum.
- Tailoring GDM care to include flexible dietary strategies and integrated psychological support may help mitigate the risk of disordered eating.

## 1 INTRODUCTION

Pregnancy involves significant physiological and psychological change, during which women may be vulnerable to a range of metabolic and mental health challenges. Conditions such as gestational diabetes mellitus (GDM) and disordered eating behaviours (DEBs) are common, but the impact of their intersection is poorly understood.

GDM is defined as hyperglycaemia first recognised in pregnancy, which usually resolves after birth and affects approximately 16% of pregnancies globally.^1^ Management of GDM typically involves modification to diet and physical activity, glucose monitoring, and in some cases pharmacological treatment.^2^ While these approaches are effective in optimising glycaemic control and improving pregnancy outcomes when properly implemented,^3^ they are psychologically and physically demanding on individuals. For some, these demands may heighten vulnerability to eating‐related distress.^4^ DEBs are maladaptive eating patterns, such as restrictive dieting, binge eating, or purging, that do not meet the full diagnostic criteria for an eating disorder (ED) but still carry significant physical and psychological risks.^5^ EDs are characterised by persistent disturbances in eating or eating‐related behaviours that significantly impair physical health or psychosocial functioning.^6^ Pregnancy represents a uniquely vulnerable period for the onset or relapse of EDs and DEBs. Physiological changes such as rapid weight gain, altered appetite and shifting body composition can be distressing for women with a history of EDs or DEBs.^7^, ^8^, ^9^

The overlap between GDM and DEBs is an emerging area of research. High incidence of DEBs has been documented in people living with type 2 diabetes mellitus (T2DM),^10^, ^11^ and both GDM and T2DM involve intensive attention to dietary intake, body weight and self‐management practices, which may exacerbate or precipitate DEBs, particularly among women with a prior history of disordered eating.^12^, ^13^ However, research specifically examining the intersection of GDM and disordered eating remains limited. Recent evidence from a multicentre cohort study suggests that DEBs are common among women with GDM, with binge eating being the most prevalent behaviour, while restrictive and compensatory behaviours occur less frequently.^14^ These behaviours have been associated with glycaemic instability, gestational weight changes, emotional distress, and adverse perinatal outcomes, including small‐ and large‐for‐gestational‐age infants.^14^, ^15^, ^16^ DEBs are therefore of particular relevance in pregnancy and GDM care, as they may be under‐recognised clinically while still adversely affecting nutritional intake, psychological well‐being and engagement with treatment.

Over the past decade, seeking health information online has become increasingly common, with social media emerging as a key space for communication, support, and information sharing. Online communities, such as Facebook groups, have been shown to provide informational and emotional support for people with GDM, helping fill gaps in formal care and potentially supporting self‐management goals.^17^ Both GDM and DEBs are highly stigmatised,^18^, ^19^ reinforcing barriers to discussing experiences in person, and leading to reduced disclosure or delayed help‐seeking.^20^, ^21^ Online forums that provide anonymity can reduce these barriers, allowing individuals to share experiences more openly and seek peer support.^22^ Researchers have increasingly used Reddit to explore lived experiences in areas such as mental health, chronic illness and other sensitive health behaviours,^23^, ^24^, ^25^, ^26^ yet no studies to date have explored the intersection of GDM and DEBs using Reddit data.

This study aims to address this gap by analysing textual data from Reddit to provide insight into experiences that might otherwise remain hidden and to highlight how these online spaces can inform understanding of patient behaviour and support needs.

## 2 AIM

To explore how women's experiences of GDM relate to the emergence or exacerbation of DEBs using Reddit posts.

## 3 METHODS

### 3.1 Data collection

Preliminary exploratory searches of Reddit were undertaken to familiarise the research team with the platform's structure and user interactions. Insights from these pilot searches informed the development of a targeted list of search terms aligned with the study objectives.

Data comprised user posts submitted to Reddit forums from the platform's inception through to November 12, 2024. Posts were retrieved via Reddit's application programming interface (API) using a predefined set of search terms related to GDM and DEBs. These terms included specific DEBs as well as broader language referring to food, eating, and dietary control (Appendix S1). Searches were restricted to English‐language posts and results were sorted through Reddit's own ‘Relevance’ algorithm. Country of origin could not be reliably determined nor analysed because Reddit is an international platform and users rarely disclose geographic location in a consistent or verifiable manner. A total of 3258 post URLs were generated and screened manually for relevance (YN, HC, SC). Posts were included if they referenced DEBs by women with current or previous GDM. Any posts related to other types of diabetes were excluded. Ambiguous cases were discussed by the research team, with active reflection on how individual assumptions might influence the screening decisions.

Posts (*N* = 144) including comments that met the inclusion criteria were randomised using the Excel random number generator. The dataset was then imported into NVivo 14 and Excel for management.

### 3.2 Data analysis

This study employed reflexive thematic analysis (RTA) to generate themes and subthemes from public Reddit discussion threads. Guided by Braun and Clarke's six‐step framework for qualitative RTA^27^ (Table 1), the analysis sought to provide rich and rigorous interpretations of users' lived experiences. RTA builds on this method by recognising the active role of the researcher in the analytic process, including consideration of how personal beliefs and assumptions shape theme development and knowledge production. Coding was conducted by a single researcher (YN). Analytic rigour was maintained through iterative discussions and reflexive questioning of interpretations with the entire research team (YN, EC, SC, HC, MB) throughout theme development.

**TABLE 1:** Description of thematic analysis process.^27^

Phase	Description of step
Data familiarisation and writing familiarisation notes	Transcribing data; reading and re‐reading; noting down initial codes and ideas
Systematic data coding	Coding key features of the data systematically across the data set, collating data relevant to each code
Generating initial themes for coded and collated data	Collating codes into potential themes; gathering all data relevant to each potential theme
Developing and reviewing themes	Checking if the themes work in relation to the coded extracts and the entire data set
Refining, defining and naming themes	Ongoing analysis to refine the specifics of each theme; generating clear names and definitions for each theme
Writing the report	Final opportunity for analysis. Selecting relevant and compelling codes/extracts; discussion of the analysis and relating it back to research question; producing report

### 3.3 Reflexivity

The research team included a resident doctor (YN), an obstetrician (EC), two undergraduate psychology students (SC, HC) and a research psychologist specialising in women's health (MB). Collectively, the team shared professional and research interests in women's health, with differing experiences of pregnancy both personally and through clinical work. These perspectives informed coding and theme development, with regular whole team discussions to reflect on assumptions and maintain reflexivity.

### 3.4 Ethics and dissemination

Reddit granted permission to use its API in January 2024. All data consisted of pre‐existing, publicly available posts from public subreddits, where users have agreed to Reddit's Privacy Policy and the public nature of their content.^28^ King's College London Ethics Team did not require formal ethical approval for this study. However, the research team recognised that collecting data from social media is a developing area of inquiry and took responsibility in mitigating ethical risks.^29^ This included replacing usernames with anonymised identifiers and removing any posts deleted on Reddit in line with platform policies.^30^ The term ‘women’ is used to reflect the language of the original posts, while recognising that not all people who are pregnant or give birth identify as women.

## 4 RESULTS

The search generated 3258 links containing posts from Reddit users. Of these posts, 4.4% were relevant to the research question. 144 posts and their comments were included in the final data set.

The analysis generated 3 themes, each containing between 2 and 4 subthemes (Figure 1).

**FIGURE 1:**
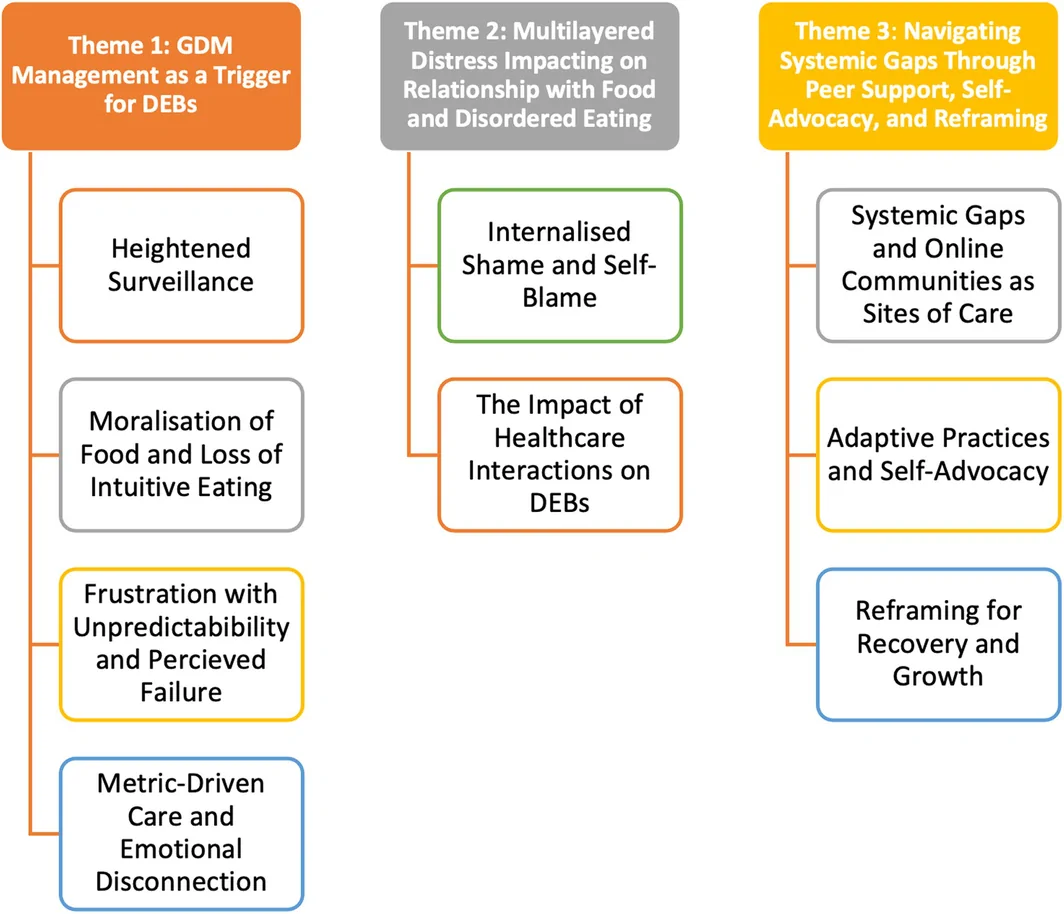
Overview of the 3 main themes and their subthemes.

### 4.1 Theme 1: GDM management as a trigger for DEBs

Women described how dietary and self‐monitoring requirements of GDM management can contribute to or exacerbate DEBs, even among those with no prior history of eating disorders.

#### 4.1.1 Heightened surveillance

Many women described developing an intense, sometimes obsessive, preoccupation with food choices and blood glucose levels. Recording carbohydrate intake and blood glucose readings amplified tendencies toward control, particularly among those with a history of disordered eating. One woman reflected on the compulsive nature of this process:

Writing down all the food I eat and having this new data to track (my blood sugar), I started feeling myself getting the same high of getting lower and lower blood sugar readings and was feeling like “what if I cut more out? how low can I get this?” before I recognized that thought process as my ED brain. (F60)

Although the dietary monitoring and glucose surveillance associated with GDM occurred during pregnancy, women frequently described the psychological and behavioural consequences of this heightened scrutiny as persisting beyond delivery and into the postpartum period.

I have developed paralyzing anxiety around eating. I have defaulted to just not eating when it is particularly bad, which is obviously unsustainable with breastfeeding and being healthy. (F95)

For others, the diagnosis reactivated previous disordered eating patterns, complicating recovery:

I've been actively recovering from BED [binge eating disorder]/bulimia … Receiving my GD diagnosis scares me … I feel like I'm crawling back into the toxicity of carb counting again. (F109)

#### 4.1.2 Moralisation of food and loss of intuitive eating

GDM management was often experienced as imposing a rigid moral framework around food, categorising dietary choices as ‘good’ or ‘bad’. This disrupted previously balanced relationships with eating and undermined recovery from prior disordered eating.

I had finally gotten to a point where I could be flexible with food and not view certain foods as good or bad, and it felt like with GD I was completely undoing all that progress. (F96)

Some women described pregnancy as an opportunity to reconnect with intuitive eating, prioritising nourishment and enjoyment without guilt. However, the GDM diagnosis reintroduced restrictive diet‐culture frameworks. Clinical guidance reinforcing ‘good’ and ‘bad’ foods heightened feelings of shame, guilt and internal conflict when dietary recommendations were not met.

Being diagnosed with GD sucked because, in pregnancy, I felt like I was eating truly intuitively for the first time, for both nutrition and for pleasure, with no fear about what it would do to my body. I really resented that now I had to “diet”. (F61)

I had an eating disorder for over 8 years and still struggle with my relationship with food, so having GD put me back into the mindset of “good versus bad” foods and made me terrified to eat anything. (F137)

#### 4.1.3 Frustration with unpredictability and perceived failure

Many women expressed frustration with the unpredictability of blood glucose fluctuations. Despite adhering to dietary recommendations, foods that were once tolerated caused unexpected glucose spikes, leading to the loss of *‘safe foods’* and feelings of failure.

…They say go for a walk after carbs, I still spike. None of it makes any sense. I eat something different than just eggs and vegetables and boom, spike. Waiting 2 h and going for a walk and being SURE the numbers are gonna be ok … and then they're high. It's so upsetting I feel like such a failure. (F42)

Even if you do everything “right” GDM is so unpredictable … I've been slowly losing “safe” foods. A little oatmeal used to be ok, and now it is 100% not. (F1)

For some, this perceived failure triggered DEBs as a way to cope with the emotional toll of managing GDM:

I felt like no matter how hard I tried I couldn't keep my number under control and what worked 1 week didn't work the next. Of course, that made me want to eat bad things or binge which was a no no. (F31)

In severe cases, unpredictability contributed to the development of EDs during pregnancy, with women reporting a fear of food and loss of trust in their bodies.

I have absolutely developed [an eating disorder]. I'm afraid of every single thing I eat, especially since I have zero consistency … Anytime I think I did good, I accidentally throw myself into ketosis for a day. (F24)

#### 4.1.4 Metric‐driven care and emotional disconnection

Women often felt that GDM management focused too heavily on glucose targets, depersonalising their care. This outcome‐driven approach reduced their experiences to mere numerical targets.

I'm a walking organic chemistry lab and my food log is my lab notebook. (F1)

Praise from clinicians for maintaining low glucose levels, while ostensibly offered as positive reinforcement, was often experienced as emotionally fraught or even distressing. For some, this validation triggered previous DEBs or psychological distress:

My doctor constantly praises me for the low numbers and how “good” I'm doing. But it doesn't feel good. At all. It feels dangerously close to when I had an eating disorder. All that anxiety and depression is coming with it, but my doctor doesn't care as long as my numbers are low. (F95)

For some women, praise for glucose control was interpreted as permission to continue restrictive eating or compensatory behaviours, such as excessive exercise.

I'm developing some really disordered behaviour like tonight after putting my toddler down, immediately doing what I can only describe as “hate” exercise, working out to near max exertion all because of that damn number. (F1)

### 4.2 Theme 2: Multilayered distress impacting on relationship with food and disordered eating

While Theme 1 highlights how the practical requirements of GDM management can trigger or exacerbate DEBs, Theme 2 focuses on the emotional and relational experiences surrounding GDM. Women described considerable distress centred on their diagnosis and pregnancy experience more broadly. This distress was shaped not only by pre‐existing vulnerabilities but also by healthcare interactions, societal expectations and fear of harming the baby. Internalised shame, guilt and exposure to fear‐based or dismissive messaging heightened food‐related anxiety and contributed to restrictive, avoidant, or compensatory eating behaviours, illustrating the emotional pathways through which GDM influences disordered eating, beyond the mechanics of management.

#### 4.2.1 Internalised shame and self‐blame

Women described how GDM evoked powerful feelings of guilt and self‐blame, particularly when food choices were perceived as harming the baby.

I feel like I'm no longer enjoying my pregnancy because I'm constantly worried about what I'm eating and feeling like I'm constantly starving plus feeling like I've somehow failed my baby. (F37)

Others described how this guilt shaped eating patterns, even leading to avoidance of healthy foods:

I'm terrified of eating because of spiking my blood sugars and hurting my baby, I'm eating way less than usual and even though I want to eat fresh, healthy things like vegetables and fruit I'm avoiding them…. (F126)

In this account, fear of harming the baby drove restrictive intake and avoidance, demonstrating how shame and guilt translated directly into DEBs.

#### 4.2.2 The impact of healthcare interactions on DEBs

DEBs were not only influenced by internal shame but also by interactions with healthcare professionals. Women felt judged or blamed during appointments, particularly regarding weight and food choices. Some reported restricting intake pre‐emptively or concealing information:

I knew then it was time to change obgyn as I am under a lot of stress each time he weight shaming me. Even months before the test. I even purposefully ate breakfast in the smallest amount possible in order for the scale to not go up every time I have to see him. (F140)

[A nurse] really made me feel quite ashamed and self‐blaming … It makes me not want to report things accurately. (F125)

Fear‐based clinical messaging emphasising severe consequences for the baby if glucose targets were not met, further increased anxiety and drove rigid, compensatory behaviours such as punitive exercise. In these ways, healthcare encounters and institutional practices acted as relational and structural triggers for DEBs, reinforcing restrictive or avoidant patterns that undermined both well‐being and glycaemic control.

Every single … meal was a roller coaster of emotions … the dichotomy of “it's not your fault” with “if you can't control this, you will harm your baby,” the shame, the guilt, the exercise as punishment … It takes such a toll. (F114)

### 4.3 Theme 3: Navigating systemic gaps through peer support, self‐advocacy, and reframing

Women's experiences highlighted significant variability in how healthcare systems addressed the intersection of GDM and disordered eating. Many described systemic gaps in care, with histories of disordered eating often minimised and mental health concerns deprioritised relative to glucose management. This perceived dismissal reinforced the sense that emotional well‐being was secondary, compounding recovery challenges and prompting some women to seek support outside clinical settings.

#### 4.3.1 Systemic gaps and online communities as sites of care

Many women described feeling overlooked within formal healthcare structures, with limited understanding or tailored interventions for their needs. Histories of disordered eating were often minimised, and mental health concerns were deprioritised relative to glucose management.

I even mentioned that [my ED] to my endocrinologist and she said “we try not to touch on that too much”. (F101)

SO MANY WOMEN have histories of disordered eating—doctors and dieticians should assume this is triggering rather than needing to be told over and over and over again. I have to tell them that they can't weigh me every single time and every single time they have a little reaction like I insulted them. (F60)

In response to these systemic gaps, some users turned to online forums for education, community, and emotional validation:

I taught myself basically everything about GD from this sub and Google. It is highly frustrating to not feel cared for when going through something that feels—and IS!—so serious. (F73)

The shared experiences of others, and especially peer‐validated research, offered a sense of legitimacy often absent in clinical settings. Reddit users sought out scientific research to support their experiences:

I found this research study so comforting in the fact that it really validates the fact that women's mental health is widely neglected as part of treatment for GDM. (F126)

#### 4.3.2 Adaptive practices and self‐advocacy

While gaps in care were common, some women reported more positive experiences when they proactively disclosed a current or past eating disorder or advocated for their needs. Early disclosure sometimes led to more flexible, psychologically sensitive care, such as reduced numerical tracking, adjusted weight monitoring, or remote support for glucose management

I also opened up about this [prior ED] with my nurses and dietician, who advised me to not log foods (when I'm not experimenting), and sometimes we have also skipped the weigh‐ ins. (F137)

I will say what helped me was specifically working with a dietician who knew my history and who is using the same sort of meal plan I did in heavy treatment—with no numbers ever mentioned … Additionally they've noted if any metal health issues arise with checking blood sugars they will argue with insurance to cover a CGM [Continuous Glucose Monitoring] and monitor from afar to allow me to not have to track as much as possible … knowing I have this support as a possibility has significantly improved my mental health! (F61)

Some women adapted further management strategies to reduce anxiety and rigidity. Adopting the “plate method” allowed some to manage GDM without strict carb counting:

I personally found it helpful to be open about my struggles with my diabetologist and now my GD is still fully diet/exercise controlled without me counting any carbs. We use the plate method and I had some time to figure out what works well for me but it's a relief that I don't have to weigh out any ingredients specifically. (F14)

Starting medication such as insulin also relieved restrictive pressure. It enabled the reintroduction of previously excluded foods and restored a sense of normality:

I was getting more and more stressed out and disordered with my own eating until I started insulin … I had been cutting out even healthy carbs like fruit and whole grains to try and make my numbers but insulin let me add those back. (F75)

#### 4.3.3 Reframing for recovery and growth

Some women engaged in cognitive reframing to create a sense of purpose or growth from their experience. Pregnancy itself was described as a motivating force for healthier eating, not as weight control, but as a means of protecting the baby:

I also suffer with disorder[ed] eating in the form of restricting calories and carbs. I do better while pregnant because someone is counting on me to get the nutrients they need. (F109)

Some participants identified GDM as a catalyst for lasting improvements in their eating behaviours. While these reflections did not negate the distress of the diagnosis, they suggested a parallel experience in which GDM prompted greater awareness of dietary habits:

GDM is super hard but it changed my lifestyle for the better. I didn't eat unhealthily but I could've eaten better. I do now. (F15)

These narratives were often contingent on access to therapeutic support. One woman described how combining GDM dietary structure with guidance from her therapist allowed her to repurpose restriction as mindful eating. This offered a framework for stabilising binge‐restrict cycles:

I've used this diet to practice eating mindfully and in moderation, with the guidance/encouragement of my therapist. I hope I can continue that after delivery. (F48)

In this way, GDM was not always experienced as wholly negative; for some, it became an unexpected opportunity to rebuild more mindful eating habits. When paired with therapeutic input, the rigidity of GDM management was at times reworked to help women overcome old patterns of bingeing or restriction. However, such positive reframing was uncommon and largely contingent on access to psychological support.

## 5 DISCUSSION

Our findings extend emerging literature linking GDM with disordered eating. We demonstrate that women explicitly describe GDM management as triggering or reactivating DEBs. In our study, Reddit users described intense preoccupation with tracking, moralisation of food, and emotional distress when targets were not met, including relapse or emergence of DEBs both during pregnancy and postpartum. These findings align with literature on T2DM where structured dietary protocols, frequent glucose monitoring, and self‐surveillance are known risk factors for DEBs in T2DM management.^11^, ^31^, ^32^ Rigid management may also amplify distress linked to perceived failure reinforcing DEBs.^33^ These findings highlight the importance of the timing of DEB risk, with heightened vulnerability during pregnancy due to intensive monitoring and dietary restrictions, and again in the postpartum period, when weight changes, societal pressures and reduced clinical support may precipitate disordered eating behaviours.

Our study highlights the significant role of systemic factors in the development of DEBs among women with GDM, suggesting that these risks arise not only from individual clinician interactions but from how GDM care is structured, monitored and evaluated. Interactions that framed food choices in moral terms or reduced management to numerical outcomes, such as blood glucose or weight, were particularly harmful. Such approaches can inadvertently reinforce disordered eating patterns through unintended clinical praise. These findings mirror emerging evidence in diabetes research that rigid dietary rules, self‐surveillance, and strict numerical targets are linked to psychological distress and disordered eating patterns.^34^, ^35^ Gaps in available support also led women to turn to online communities for advice, validation, and shared strategies. While peer support can foster self‐efficacy, as seen in studies of Facebook groups for GDM,^17^ reliance on unmoderated spaces such as Reddit also raises the risk of misinformation, highlighting the need for clinical pathways that proactively address women's psychological needs and disordered eating risk.

Our findings also highlight the postpartum period as a particularly high‐risk window for the re‐emergence of DEBs. Reddit users described relapse behaviours such as bingeing, restriction, and heightened anxiety following GDM, echoing qualitative research showing that EDs frequently re‐emerge postpartum, driven by self‐criticism, societal pressures, and healthcare messaging.^36^ It is important to consider that most prior studies were conducted in high‐income countries, highlighting the need to consider cultural and healthcare system contexts in interpreting postpartum risk. Despite this, some women reframed GDM management positively, especially with therapeutic support, viewing dietary frameworks as opportunities for mindful eating or promoting well‐being for themselves and their baby. Similar findings have been reported in type 1 diabetes mellitus (T1DM), where individualised care and psychological support improve treatment attitudes and overall well‐being.^37^ These accounts underscore the value of tailored approaches that integrate dietary guidance with psychological support, helping women engage with GDM care in ways that reduce distress and mitigate disordered eating risk.

Women described CGM as allowing them to outsource glucose tracking to their healthcare team, reducing the need to engage in strict tracking behaviours. This suggests such technologies may both enhance metabolic control and potentially buffer against DEBs. CGM has been shown to reduce fear of hypoglycaemia and improve treatment satisfaction in adults,^38^ increase hypoglycaemia confidence in young people,^39^ and alleviate hypoglycaemia‐related anxiety in pregnant women with T1DM.^40^ However, CGM‐related distress has been documented, highlighting the need to consider its psychological impact and the risk of it becoming another focal point for individuals prone to DEBs.^41^ Visual tools such as the “plate method” may serve as a complementary strategy to reduce rigidity without compromising glycaemic control. This approach, which visually divides the plate into vegetables, protein, and carbohydrates, allows women to manage meals flexibly without strict numerical tracking, supporting both metabolic and psychological outcomes.^42^

In recent years, Reddit has emerged as a valuable social media platform for examining public discourse on both mental and physical health conditions. To our knowledge, no previous studies have explored discussions of GDM and DEBs on social media platforms. While our findings cannot capture the full range of concerns experienced by women with GDM, they highlight issues voiced in peer‐to‐peer exchanges. This is particularly relevant given the growing reliance on social media for medical advice, where information is not subject to clinical oversight and may amplify misinformation or distress. Understanding these narratives is therefore crucial for healthcare professionals, not only to appreciate the support women seek outside clinical settings, but also to identify gaps in care that may drive women to online communities.^43^

### 5.1 Strengths and limitations

A strength of this study lies in its use of the Reddit social media platform, allowing access to candid narratives that might not emerge in conventional research due to stigma or fear of judgement. The anonymity of the platform may have facilitated disclosure of sensitive experiences related to food, pregnancy, and mental health. However, this same anonymity precludes verification of user identities, demographic characteristics, or medical histories. The dataset was restricted to posts written in the English language, publicly available posts, limiting transferability across cultural contexts. Although users' geographical locations could not be verified, the language used, healthcare references and care pathways described in posts suggested that many contributors were likely based in high‐income countries. This may further limit the transferability of findings to low‐ and middle‐income settings with different models of GDM care. Furthermore, no validated diagnostic tools were applied, meaning the presence and severity of DEBs could not be confirmed. Posts rarely included consistent demographic information such as body mass index (BMI), limiting exploration of whether experiences differed by body size or weight status. Finally, while reflexive analysis enhanced the interpretive depth of the analysis, the absence of perspectives from individuals with lived experience of GDM and disordered eating may have limited the range of viewpoints considered in the analysis and interpretation of results.

## 6 CONCLUSION

Aspects of GDM management, including intensive monitoring, dietary restriction, and a focus on numeric targets, may increase the risk of DEBs or exacerbate distress for some women. Our findings highlight how moralised messaging and fear‐based communication can increase anxiety and disrupt relationships with food. At the same time, limited psychological support and fragmented care pathways leave many women to seek understanding and validation online. These findings highlight the need for further research into women's experiences with GDM to guide care that supports metabolic health while minimising the risk of DEBs. Longitudinal studies could explore how eating‐related distress evolves across pregnancy and the postpartum period, including differences between women with current versus prior DEBs, in order to further inform how care protocols might be adapted to reduce DEB risk.

## FUNDING INFORMATION

The Study is part funded by the National Institute for Health and Care Research (NIHR) through an Advanced Fellowship awarded to Madeleine Benton (ref: NIHR304430). The views expressed are those of the author(s) and not necessarily those of the NIHR or the Department of Health and Social Care.

## CONFLICT OF INTEREST STATEMENT

The authors disclose no conflict of interest relating to this study.

## ETHICS STATEMENT

Not applicable.

## Supporting information


Appendix S1.

## Data Availability

As the dataset comprises screenshots of Reddit posts imported into NVivo, the full data cannot be shared due to the risk of user identifiability. De‐identified excerpts used in the analysis are presented within the manuscript, and anonymised coding documentation is available from the corresponding author upon reasonable request.
