# Supplementary material for: Gestational diabetes mellitus and disordered eating behaviours: A qualitative study of online forum discussions
Source: Diabet Med. 2026 Apr 17;43(10):e70322. doi: 10.1111/dme.70322 (PMC13643613; doi:10.1111/dme.70322)
Supplement: Supplementary file 1 [file DME-43-e70322-s001.docx]

**Appendices**

***Appendix 1: Key Search Terms***

| **Concept 1**  **GDM** | **AND** | **Concept 2**  **Disordered Eating** | |
| --- | --- | --- | --- |
| Gestational Diabetes Mellitus  Gestational Diabetes  GDM  GD  Gest Diabetes  Diabetes pregnancy (exclude T1DM, T2DM) |  | Disordered eating  Eating disorder  ED  Eating behavio(u)r  Restricting/Restrict  Binge/Binging/Binged  Anorexia/Anorexic  Bulimia/Bulimic  Starve/Starved Starving)  Purge/Purging  Eating  Food  Diet  Weight | Calorie(s)  Carbs  Carbohydrate  Compulsive eating  Obsessive/obsessed/obsessing  Tracking  Counting  Triggering/Triggered  Control/Controlling/Controlled  Food anxieties/anxiety |
